# MCL-SWT: Mirror Contrastive Learning with Sliding Window Transformer for Subject-Independent EEG Recognition †

**DOI:** 10.3390/brainsci15050460

**Published:** 2025-04-27

**Authors:** Qi Mao, Hongke Zhu, Wenyao Yan, Yu Zhao, Xinhong Hei, Jing Luo

**Affiliations:** 1 School of Data Science and Engineering, Xi’an Innovation College of Yanan University, Xi’an 710100, China; maoqi_xiancxxy@163.com (Q.M.);; 2 Human-Machine Integration Intelligent Robot Shaanxi University Engineering Research Center, School of Computer Science and Engineering, Xi’an University of Technology, Xi’an 710048, China

**Keywords:** BCI, EEG, motor imagery, contrastive learning, sliding window transformer

## Abstract

**Background**: In brain–computer interfaces (BCIs), transformer-based models have found extensive application in motor imagery (MI)-based EEG signal recognition. However, for subject-independent EEG recognition, these models face challenges: low sensitivity to spatial dynamics of neural activity and difficulty balancing high temporal resolution features with manageable computational complexity. The overarching objective is to address these critical issues. **Methods**: We introduce Mirror Contrastive Learning with Sliding Window Transformer (MCL-SWT). Inspired by left/right hand motor imagery inducing event-related desynchronization (ERD) in the contralateral sensorimotor cortex, we develop a mirror contrastive loss function. It segregates feature spaces of EEG signals from contralateral ERD locations while curtailing variability in signals sharing similar ERD locations. The Sliding Window Transformer computes self-attention scores over high temporal resolution features, enabling efficient capture of global temporal dependencies. **Results**: Evaluated on benchmark datasets for subject-independent MI EEG recognition, MCL-SWT achieves classification accuracies of 66.48% and 75.62%, outperforming State-of-the-Art models by 2.82% and 2.17%, respectively. Ablation studies validate the efficacy of both the mirror contrastive loss and sliding window mechanism. **Conclusions**: These findings underscore MCL-SWT’s potential as a robust, interpretable framework for subject-independent EEG recognition. By addressing existing challenges, MCL-SWT could significantly advance BCI technology development.

## 1. Introduction

Brain–computer interface (BCI) is a human–computer interaction technology that enables information exchange and control between the brain and external devices. It has received extensive attention in the fields of brain science and artificial intelligence [1,2]. BCI systems operate by translating brain activity into information or commands that devices can understand or execute, thereby facilitating the connection between an individual’s mind and the external world [3]. According to the different ways of capturing signals, BCI technologies can be classified into invasive BCI and non-invasive BCI [4]. An ethical issue often encountered with BCI as an assistive technology is that while BCI can improve the quality of life for people with disabilities and their loved ones, BCI devices may increase the stigma of disability associated with the individual, which may influence potential users not to use BCI [5]. Non-invasive BCI refers to BCI technology that does not require surgery or direct contact with brain tissue, such as Electroencephalography (EEG). Motor imagery (MI) is a crucial paradigm in the development of BCI. The MI paradigm in BCI involves the mental imagery of limb movements or other body parts, allowing users to control external devices or perform specific tasks through mental imagery, which has been applied in post-stroke motor function rehabilitation [6,7]. The MI EEG captures EEG signals during MI tasks, which is convenient, simple, flexible, non-invasive, and minimally demanding on the environment [8]. Achieving accurate decoding of EEG signals is crucial for the development of stable MI-BCI-based rehabilitation systems.

With the advancement of machine learning technologies, many machine learning algorithms have been applied to EEG signal decoding [9]. For example, linear discriminant analysis (LDA) [10] and support vector machine (SVM) [11], but these methods require the manual design of feature extraction methods. As an improved variant of CSP [12], the Filter Bank Common Spatial Pattern (FBCSP) [13] overcomes the limitation of relying on a single specific frequency band and further enhances performance through a multi-band analysis strategy. End-to-end feature extraction and classification methods based on CNNs have shown excellent performance in the field of MI-BCI [6,14,15]. Schirrmeister et al. explored the impact of different CNN model designs on the classification of EEG signals from MI [16]. Although these methods perform well in subject-dependent classification tasks, their performance in subject-independent scenarios still requires improvement. Notably, most current MI-based BCI applications rely on subject-dependent settings, which poses significant challenges in practical implementations: on the one hand, each new subject requires the collection of a substantial amount of individual data for model calibration. On the other hand, classification models designed for specific subjects often perform poorly on other subjects [17,18]. This undoubtedly increases system complexity while reducing practicality and real-time performance. Therefore, the research of subject-independent BCI systems focuses on solving the problem of generalization between subjects, based on which BCI systems can effectively adapt to multiple subjects, expanding their scope of application and enhancing their adaptability.

A decrease in frequency energy in relevant areas of the cerebral cortex is known as event-related desynchronization (ERD), and an increase in frequency energy is known as event-related synchronization (ERS) [19]. ERD/ERS in the contralateral sensorimotor cortex was analyzed using standardized electroencephalographic protocols. The baseline mu-rhythm power (8–13 Hz frequency range) was established during the pre-movement interval from 500 ms to 100 ms before movement onset. Post-movement cortical dynamics were evaluated through non-overlapping 300 ms epochs spanning 0–1500 ms after movement initiation. ERD/ERS magnitude was quantified as the percentage power decrease relative to baseline, with statistical significance assessed through cluster-based permutation testing (5000 iterations) to address multiple comparison challenges. ERD and ERS exhibit spatial variability across subjects, and Independent Component Analysis (ICA) [20] is commonly employed to mitigate this spatial variability [21]. Kim et al. [21] extracted ERD/ERS using EEG-independent electron sources obtained by ICA and used them for ERSP-based movement classification. Zhao et al. [22] proposed an end-to-end deep domain adaptation method to improve the classification performance of a single subject using valid information from multiple subjects (source domains) in order to address the significant differences between subjects. Xu et al. [23] proposed an online pre-alignment strategy based on Riemannian Procrustes Analysis (RPA) [24] for aligning the EEG distributions of different subjects prior to the training and inference process, thereby reducing cross-dataset variability. Traditional BCI classification utilizes discriminative features representing ERD/ERS to classify MI [25]. Most methods use a transfer learning strategy to address inter-subject differences. MI of the left or right hand induces ERD in the brain’s contralateral sensory areas and simultaneously induces ERS in the ipsilateral sensory areas [19]. Therefore, monitoring the locations of ERD/ERS phenomenon in the brain’s sensorimotor areas provides an important foundation for classifying MI EEG signals. The spatial variance in ERD/ERS phenomenon across different subjects presents significant research challenges [25]. Consequently, improving the model’s ability in recognizing ERD and ERS is crucial for enhancing the performance of subject-independent MI EEG signal recognition.

While deep learning techniques have made significant advancements in MI based EEG signal recognition, these models are often viewed as black boxes. Their decision-making processes are difficult to interpret and comprehend, leaving us uncertain whether they are grounded in the identification of ERD/ERS patterns within the sensorimotor regions of the brain, which is a key factor in classifying MI. In addition, the success of the transformer model in natural language processing and computer vision has drawn increasing attention from BCI researchers. The attention mechanism has become a focal point in research on MI EEG decoding, demonstrating a strong ability to extract discriminative features from EEG signals [8,26,27]. Luo et al. proposed a shallow Transformer model to capture discriminative segments in multi-subject EEG signals through a multi-head self-attention mechanism to improve the classification accuracy of MI tasks [8]. Altaheri et al. designed an attention-based temporal convolutional network (ATCNet) model, which combines a multi-head self-attention mechanism and a spatio-temporal convolutional network to efficiently extract the spatio-temporal features of MI-EEG and improve the classification performance with fewer parameters [28]. But the computational complexity of the global multi-head self-attention mechanism in transformer models increases quadratically with the length of the input sequence. At present, most transformer models applied in MI-EEG recognition utilize short input sequences, which restricts the temporal resolution of the features obtained [27].

To address the issues mentioned above, we propose a mirror contrastive learning-based sliding window transformer (MCL-SWT) to enhance subject-independent MI-based EEG signal recognition. This manuscript is a conference extended paper, and the original paper was published in [29].

The main contributions of this paper are as follows:

(1) According to research in the field of neurology, mental imagery of left or right-hand movements induces ERD in the contralateral sensorimotor regions of the brain. Based on this finding, this study introduces mirror contrastive learning (MCL), which enhances the accuracy of identifying the spatial distribution of ERD/ERS by contrasting the original EEG signals with their mirror EEG signals obtained by exchanging the channels of the left and right hemispheres.
(2) For subject-independent EEG recognition, a temporal sliding window transformer (SWT) is proposed to achieve high temporal resolution in feature extraction while maintaining manageable computational complexity. Specifically, the self-attention scores are computed within temporal windows, and as these windows slide along the EEG signal’s temporal dimension, the information from different temporal windows can interact with each other.
(3) The experimental results on subject-independent based MI EEG signal recognition demonstrate the effectiveness of MCL-SWT method. Parameter sensitivity experiments demonstrated the robustness of the MCL-SWT model, and feature visualization and ablation studies further validated the effectiveness of the MCL method.

## 2. Method

**Figure 1:**
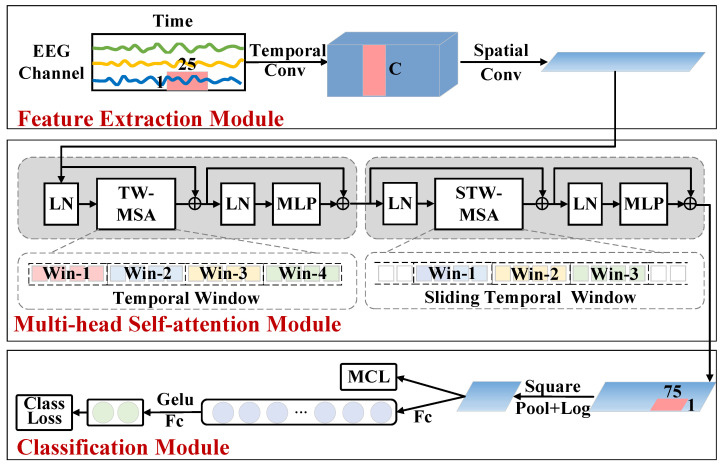
Illustration of the overall architecture of the MCL-SWT model.

This section provides the detailed description of the MCL-SWT model, and the framework of the MCL-SWT model is illustrated in Figure 1.

### 2.1. Notations and Definitions

The EEG data collected in a session is defined as ${\left\{\left(X_{i},Y_{i}\right)\right\}}_{i=1}^{n}$, where *n* represents the number of EEG trials. $X\in R^{T\times C}$ denotes the EEG trial, *T* is the number of sampling points and *C* is the number of EEG channels. The raw EEG signal *X* serves as the input of the MCL-SWT model, with a batch size of *B*, resulting in an input data dimension of B×1×T×C.

### 2.2. Sliding Window Transformer Model

For subject-independent EEG recognition, a temporal sliding window transformer (SWT) is proposed to achieve high temporal resolution in feature extraction while maintaining manageable computational complexity. The overall architecture of the SWT model consists of three parts: a feature extraction module, a multi-head self-attention module, and a classification module, as shown in Figure 1.

#### 2.2.1. Feature Extraction Module

Initially, drawing inspiration from the bandpass filters used in the filter bank common spatial pattern algorithm [13], we used a temporal convolution $C_{t}$ with a kernel size of T×1 to process temporal information. Larger convolution kernels help to achieve a wider range of signal transformations at this stage. Secondly, in order to simulate the spatial filter in the common spatial pattern algorithm, a spatial convolutional layer $C_{s}$ with a convolutional kernel size of 1×C is used to fuse the individual channels of the EEG. Then, a batch normalization layer BN is added, which is a technique used to normalize the model parameters with the aim of increasing the training speed of the neural network model, improving the convergence of the model, and reducing the occurrence of overfitting [30]. This CNN-based feature extraction module is able to learn shallow features with spatio-temporal information and is able to reduce the dimensionality of the shallow spatio-temporal features, thus reducing the amount of computation required to perform the self-attention computation afterwards, which can be described as:

(1) $$F=\mathrm{BN}\left(C_{s}\left(C_{t}(X)\right)\right)$$

#### 2.2.2. Multi-Head Self-Attention Module

**Figure 2:**
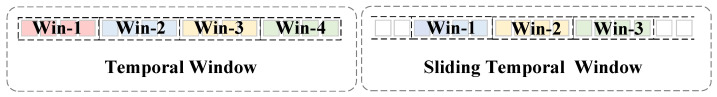
Illustration of temporal window and sliding temporal window.

A multi-head self-attention module based on temporal sliding window is introduced to capture the global temporal dependencies of EEG features. This module consists of two blocks: a multi-head self-attention block based on a temporal window and another based on a sliding temporal window, with the primary distinction being the segmentation of the temporal window, as illustrated in Figure 2. The different segmentations of the temporal window enable effective information exchange between sequences in neighboring windows.

Each multi-head self-attention block incorporates a sequence of components: a layer normalization (LN), a temporal window-based multi-head self-attention layer (TW-MSA), a layer normalization, and a multi-layer perceptron layer (MLP), along with two additional residual network structures to enhance feature learning and stability. Each layer is detailed as follows:

(1) LN: The LN layer performs normalization along the feature dimensions of each sample, ensuring that the output features are normalized as described in [31]. This normalization enhances the network’s ability to learn and generalize, particularly when handling data with complex feature distributions.

(2) TW-MSA: The TW-MSA layer is designed to capture local temporal dependencies by computing self-attention scores within a specified temporal window. As illustrated in Figure 1, the attention score computation is restricted to each local window, which improves the model’s sensitivity to local information while enhancing computational efficiency. Based on empirical evidence, the window size for TW-MSA is set to 8. Additionally, a residual connection is incorporated alongside the LN and TW-MSA layers to promote stable gradient flow and model optimization. The specific calculation steps for TW-MSA are as follows:

(2) $$Q_{i}=W_{i}^{Q}\cdot \mathrm{LN}\left(F\right)$$

$$K_{i}=W_{i}^{K}\cdot \mathrm{LN}\left(F\right)$$

$$V_{i}=W_{i}^{V}\cdot \mathrm{LN}\left(F\right)$$

(3) $$h_{i}=\mathrm{Attention}\left(Q_{i},K_{i},V_{i}\right)=\mathrm{Softmax}\left(\frac{Q_{i}K_{i}^{T}}{\sqrt{d_{k}}}\right)V_{i}$$

(4) $$O=\mathrm{concat}\left(h_{1},\cdots ,h_{H}\right)W^{O}+F$$

where $W_{i}^{Q}\in R^{d_{\mathrm{model}\;}\times d_{q}},W_{i}^{K}\in R^{d_{\mathrm{model}}\times d_{k}},W_{i}^{V}\in R^{d_{\mathrm{model}}\times d_{v}}$ and $W^{O}\in R^{\left(H\cdot d_{v}\right)\times d_{\mathrm{model}}}$ are the corresponding weight matrices, $h_{i}$ represents the *i*-th attention head, *H* denotes the number of attention heads, $d_{\mathrm{model}\;}$ is the dimension of the input embedding, and $d_{q},d_{k},d_{v}$ represent the dimensions of the query, key, and value, respectively.

(3) MLP: After the second LN, an MLP is added, which consists of a positionally fully connected layer (FC), a GELU activation function (G) [32] and another positionally fully connected layer. Additionally, a residual connection is incorporated alongside the LN and MLP layers to promote stable gradient flow and model optimization. The MLP layer is used to perform further nonlinear transformations and feature extraction on the attentional outputs to enhance the representation and fitting ability of the model, and also to increase the depth of the model’s network so that the model is able to learn deeper levels of abstract features.

(5) A=O+FC(G(FC(LN(O))))

To enhance the correlation between local windows and capture the global temporal dependencies of EEG signals, another multi-head self-attention block based on sliding temporal window is added after the first multi-head self-attention block. This block facilitates information interaction by cyclically shifting local windows. As illustrated in Figure 2, the local window is shifted to the right in steps of half the window size, M/2, and self-attention scores are then computed within the newly formed temporal windows. By integrating two multi-head self-attention block, the model reduces the computational cost of attention weight calculations while significantly improving its ability to model long-range temporal dependencies. This combination effectively enhances both the efficiency and performance of the model.

The computational complexity of the global multi-head self-attention mechanism is quadratic in the length of the input vectors, as it is necessary to compute the attentional weights between each input position:

(6) $$\Omega \left(MSA\right)=4LD^{2}+2L^{2}D$$

where *L* is the input vector length and *D* is the input dimension. Whereas in multi-head self attention model with sliding window (WMSA), the window size is fixed to *M*, then the computational complexity is linearly related to the input vector length.

(7) $$\Omega \left(WMSA\right)=8LD^{2}+4MLD$$

Therefore, when the input vector length is much larger than the window size, multi-head self attention model with sliding window has an obvious advantage in reducing the computational complexity.

#### 2.2.3. Classification Module

A square non-linear function is first added to the classification module for activation. Then, an average pooling layer is applied to reduce the temporal feature dimensions, after which a log activation function is added. Then, the EEG features with global temporal dependencies are input into the fully connected layer for classification, and finally a SoftMax function is applied to compute the predicted probabilities. The category with the highest predicted probability value is selected as the classification result of the EEG signals. As the output of the multi-head self-attention module is *A*, the output of the classification module is:

(8) P=Softmax(FC(log(AvgPool(A∘A))))

Finally, the predicted probabilities for the mirror EEG signals (described below) corresponding to left MI and right MI are swapped, and then added to the predicted probabilities of the original EEG trial to obtain the final predicted probabilities, as described in [8]:

(9) $$\left[p_{l},p_{r}\right]=\left[p_{l}^{o}+p_{r}^{o}\right]+\left[p_{r}^{m}+p_{l}^{m}\right]$$

### 2.3. Mirror Contrastive Learning

In EEG signals, when people perform left or right hand MI, ERD occurs in the contralateral sensorimotor region of the brain, while ERS occurs in the ipsilateral region. Therefore, monitoring the locations of ERD/ERS phenomenon in the sensory regions of the brain is an important basis for classifying MI EEG signals. However, existing deep learning models function primarily as black boxes, making it challenging to identify ERD/ERS phenomenon, which presents significant challenges for research. To address this issue and enhance the localization ability of MI recognition models, we propose mirror contrastive learning. The MCL increases the distance in feature space between EEG signals containing ERD from different sides of the sensorimotor cortex (negative samples pair), while reducing the distance between EEG signals containing ERD from the same side of the sensorimotor cortex (positive samples pair). In this way, the model learns to effectively locate the ERD phenomenon.

#### 2.3.1. Mirror EEG Signal

**Figure 3:**
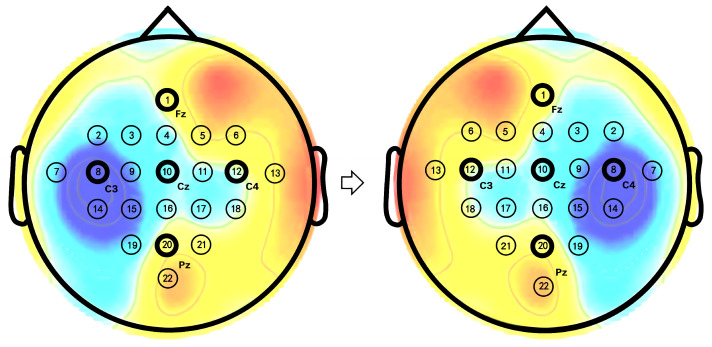
Mirror EEG signals are created to generate negative samples pair for MCL.

The mirror EEG signal is constructed to increase the number of negative sample pairs in the mirror contrastive learning. This is achieved by swapping the EEG channels between the right and left brain hemisphere regions to create the mirror EEG signals [8,33], as shown in Figure 3. For instance, the data from the original C3 channel is exchanged with the C4 channel, and vice versa. The electrode positions of the mirror EEG signals are the same as those of the original EEG signals in a mirror, hence the term “mirror EEG signals”. This approach ensures that the ERD/ERS phenomenon appears on the contralateral side when comparing the mirror EEG signal with the original EEG signal. Additionally, it is crucial to assign the opposite label to the mirror EEG signal relative to the original EEG signal. For example, if the original EEG label is “left hand”, the label for the mirror EEG signal will be “right hand”.

Initially, both the mirror and original EEG signals are used in the training process, effectively doubling the number of training samples through this data augmentation technique. Simultaneously, the mirror and original EEG signals are paired as negative samples and applied in the mirror contrastive loss function.

#### 2.3.2. Mirror Contrastive Loss Function

**Figure 4:**
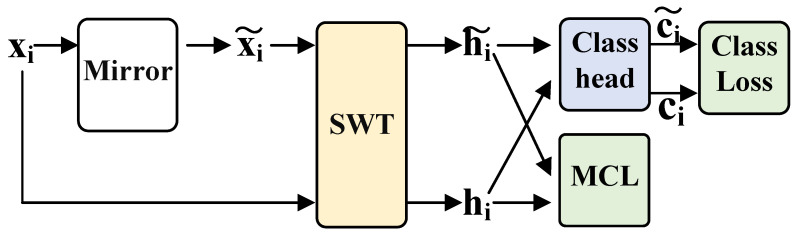
MCL-SWT Model training framework.

As described in the literature [34], the contrastive loss method guides the model to optimize the feature representation during training by comparing positive sample pairs (similar features) with negative sample pairs (dissimilar features). Specifically, the model is encouraged to bring the features of the positive sample pairs closer together while pushing the features of the negative sample pairs farther apart. However, the successful construction of positive and negative sample pairs is an important factor in contrast loss. By constructing mirror EEG signals, we apply the mirror contrastive loss to enhance the model’s sensitivity to ERD/ERS locations by contrasting the original EEG signals with their corresponding mirror EEG signals as shown in Figure 4.

As in conventional contrastive learning methods, samples originating from the same MI task category are treated as positive sample pairs, while those originating from different MI task categories are treated as negative sample pairs in this study:

(10) $$L_{o}=\sum_{i,j\in OE}\max\left(\left(\alpha +g_{ij}\left(D_{ij}-\beta \right)\right),0\right)$$

where α controls the degree of separation between positive sample pairs, while β determines the boundary between positive and negative sample pairs. $D_{ij}$ represents the Euclidean distance between the pair of samples *i* and *j*. The variable $g_{ij}$ is assigned a value of 1 for positive sample pairs and −1 for negative sample pairs. And OE denote the original EEG signal set. This loss function offers robustness to noise since it permits a certain degree of tolerance and does not overly penalize the model for minor similarities arising from noise.

Additionally, to emphasize the distinct locations of the ERD/ERS phenomenon between the original EEG signal and its mirror counterpart, an additional contrastive loss is applied to the sample pairs formed by the original EEG signal and mirror EEG signal, as follows:

(11) $$L_{m}=\sum_{i\in ME,j\in OE}\max\left(\left(\alpha +g_{ij}\left(D_{ij}-\beta \right)\right),0\right)$$

where ME denote the mirror EEG signal set. Therefore, the mirror contrative loss function is given as:

(12) $$L_{mc}=w_{o}L_{o}+w_{m}L_{m}$$

where $w_{o}$ and $w_{m}$ denote the weights of each loss. By the application of mirror contrastive loss function in the training of model, the model’s ability to accurately localize ERD/ERS locations is enhanced by contrasting the original EEG signals with the mirror EEG signals. Specifically, the model is encouraged to push the original EEG signal and its mirrored version apart in the feature space. The mirror contrastive loss is applied to the feature after the log activation function as shown in Figure 1.

#### 2.3.3. Model Training Loss

For MI EEG recognition, the cross-entropy loss was used as the loss function:

(13) $$L_{c}=-\frac{1}{N}\sum_{i=1}^{N}y_{i}\ln\left(p_{i}\right)$$

where *N* is the total number of MI EEG trials, $y_{i}$ is the true label of the *i*th MI EEG trial, and $p_{i}$ is the probability of the *i*th MI EEG trial category predicted by the model.

Therefore, the final loss function of the model training is the sum of the mirror contrastive loss and the classification loss:

(14) $$L=L_{c}+L_{mc}$$

The training framework is given in Figure 4. To provide a clear description, we show the pseudo-code for MCL-SWT in Algorithm 1.
**Algorithm 1** The MCL-SWT Method**Input:** The training set $\left\{X,Y\right\}$, hyperparameters $\alpha ,\beta ,w_{o},w_{m}$, max number of iterations $I_{max}$.**Output:** Model parameters *W*. 1:initialize *W*. 2:**for** *i*=1 to  $I_{max}$ **do** 3:      **for** *l*=1 to $l_{d}$ **do** 4:          sample a batch of original EEG signal $\left\{\left(X^{O},Y^{O}\right)\right\}$ from set $\left\{X,Y\right\}$; 5:          construct a batch of mirror EEG signal $\left\{\left(X^{M},Y^{M}\right)\right\}$ from set $\left\{\left(X^{O},Y^{O}\right)\right\}$; 6:          calculate the training loss using (14); 7:          backpropagation calculates the error of each layer, calculates the reciprocal of the parameters *W*, and updates the parameters *W*; 8:      **end for** 9:**end for**10:**return** *W*

## 3. Experiment and Data

In this section, we describe two publicly available EEG datasets, how the data is preprocessed, and how the data is divided. A code implementation of MCL-SWT is available at https://github.com/roniusLuo/MCL_SWT (accessed on 20 April 2025).

### 3.1. Data

To evaluate our proposed method, we conducted extensive experiments on three public MI datasets, BCI Competition IV datasets 2a, 2b [35], and OpenBMI datasets [36].

(1) BCI Competition IV Dataset 2a: The BCI Competition IV Dataset 2a included the EEG signals of four-category MI recognition tasks (left hand, right hand, feet, tongue) from nine subjects. For each subject, two sessions of EEG signals were collected on different days, and there were total 288 trials (72 trials per class) per session. At the beginning of each training session, approximately 5 min of recording were conducted to assess EOG influence. The recording was divided into three segments: (1) 2 min with eyes open (gazing at a fixation cross on the screen), (2) 1 min with eyes closed, and (3) 1 min with eye movements. Subjects were seated comfortably in an armchair facing a computer screen. Each trial began (t = 0 s) with a fixation cross appearing on a black screen, accompanied by a short auditory warning tone. After 2 s (t = 2 s), a visual cue (arrow pointing left, right, down, or up-corresponding to the four classes) appeared and remained on screen for 1.25 s. No feedback was provided. Subjects were instructed to perform the motor imagery task until the fixation cross disappeared at t = 6 s.

During the EEG acquisition, according to the prompts on the screen, the subjects performed four different types of MI tasks, and 22 channels of EEG were acquired simultaneously, using electrode positions in the 10–20 international standard lead system. 22 Ag/AgCl electrodes (electrode spacing 3.5 cm) were used to record the EEG signals, and the electrode arrangement is shown in the left side of Figure 3. All signals were recorded monopolarly with the left mastoid serving as reference and the right mastoid as ground. The signals were sampled with 250 Hz and bandpass-filtered between 0.5 Hz and 100 Hz. The sensitivity of the amplifier was set to 100 μV. An additional 50 Hz notch filter was enabled to suppress line noise. In addition to the 22 EEG channels, 3 monopolar EOG channels were recorded and also sampled with 250 Hz. They were bandpass filtered between 0.5 Hz and 100 Hz (with the 50 Hz notch filter enabled), and the sensitivity of the amplifier was set to 1 mV.

(2) BCI Competition IV Dataset 2b: The EEG signals of two-class MI tasks (left hand and right hand) from another nine subjects were collected on BCI Competition IV Dataset 2b. The subjects were right-handed and had normal or corrected-to-normal vision. All volunteers were sitting in an armchair, watching a flat screen monitor placed approximately 1 m away at eye level. For each subject, five sessions are provided, whereby the first two sessions contain training data without feedback (screening), and the last three sessions were recorded with feedback. At the beginning of each session, a recording of approximately 5 min was performed to estimate the EOG influence. The recording was divided into three blocks: (1) two minutes with eyes open (looking at a fixation cross on the screen), (2) one minute with eyes closed, and (3) one minute with eye move- ments. The artifact block was divided into four sections (15 s artifacts with 5 s resting in between) and the subjects were instructed with a text on the monitor to perform either eye blinking, rolling, up-down, or left-right movements. At the beginning and at the end of each task, a low and high warning tone were presented.

Three bipolar recordings (C3, Cz, and C4) were recorded with a sampling frequency of 250 Hz. The recordings had a dynamic range of ±100 μV for the screening and ±50 μV for the feedback sessions. They were bandpass- filtered between 0.5 Hz and 100 Hz, and a notch filter at 50 Hz was enabled. The placement of the three bipolar recordings were slightly different for each subject. The electrode position Fz served as EEG ground.

(3) OpenBMI dataset: The OpenBMI dataset consisted of EEG data from 25 females and 29 males for a total of 54 subjects with an age range of 24 to 35 years. All participants had no history of neurological, psychiatric, or other relevant disorders that could affect the results of the experiment. Of these 54 subjects, 21 were normal motor imagery subjects and 33 were BCI illiteracy subjects, i.e., a group unable to use the BCI proficiently or correctly. For the first 3 s of each trial, a black gaze cross was displayed in the centre of the screen to help prepare the subject for the motor imagery task.The EEG signal was acquired at a sampling rate of 1000 Hz through 62 Ag/AgCl electrodes. Subsequently, an arrow to the right or left was displayed on the screen as a visual cue, and the subject was required to perform the corresponding hand grasping imagery task for 4 s. After each task, the screen would remain blank for 6 s (±1.5 s). The experiment was divided into a training phase and a test phase, each containing 100 trials and an equal number of right- and left-handed motor imagery tasks. The EEG amplifier used in the experiment was a BrainAmp. The channels were nasion-referenced and grounded to electrode AFz. Additionally, an EMG electrode recorded from each flexor digitorum profundus muscle with the olecranon was used for reference.

### 3.2. Data Preprocessing

(1) BCI Competition IV Dataset 2a, 2b: The experiments in this paper are based on the Braindecode EEG processing toolbox [16]. The experiments used 4.48 s EEG segments, acquired from 0.5 s before the appearance of motor imagery cues (this prompted the subjects to perform the desired motor imagery task) to 3.98 s after cue onset. In order to allow the model to autonomously perform feature learning and detect valid EEG signal segments, only minimal preprocessing was performed on the EEG signal trials. Two types of classification tasks (left- and right-handed) were first performed using the C3, Cz, and C4 EEG channels, and data from these channels were converted from volts to microvolts to improve numerical stability. Subsequently, a third order band-pass filter from 4 to 38 Hz or 0–38 Hz was used for filtering along the time axis to highlight or remove signal components in a specific frequency range, and finally using the channel exponential sliding normalization technique, the initial window size for calculating the mean/variance was calculated with a value of init_block_size set to 1000, and the value of factor_new set to 0.001. In order to maintain numerical stability, eps is set to 0.0001.

(2) OpenBMI dataset: The dataset was divided into different BCI illiteracy paradigms (people who cannot use BCIs correctly), so we excluded data from this group of subjects from the experiment, and only 21 normal MI BCI users (subject 1, subject 2, subject 3, subject 5, subject 6, subject 9, subject 17, subject 18, subject 19, subject 21, subject 22, subject 28, subject 29, subject 32, subject 33, subject 36, subject 37, subject 43, subject 44, subject 45, subject 52) retained EEG signal data for experimental purposes. In this paper, 4.48 s EEG segments with C3, C4, and Cz electrodes were selected for the experiments. The EEG signal data were first filtered along the time axis (axis = 1) using a 3rd order bandpass filter from 8 to 30 Hz to highlight or remove signal components in a specific frequency range, followed by a channel exponential sliding normalization technique to calculate the mean/variance with the initial window size of init_block_size set to 1000 and factor_new set to 0.001, in order to maintain the numerical stability, eps was set to 0.0001, and the EEG signal was resampled using a frequency of 250 Hz.

The aim of this paper is to investigate the classification performance of subject-independent MI EEG signals, and in order to achieve this goal, a cross-validation approach is used to partition the dataset to ensure that the train and test sets are from different groups of subjects. When using the train sessions of all nine subjects in dataset IIa as the train set, the test sessions of all nine subjects in dataset IIb (session 4 and session 5) were used as the test set; conversely, when using the train sessions of all nine subjects in dataset IIb (session 3) as the train set, the test sessions of all nine subjects in dataset IIa were used as the test set. By dividing the data in this way, we are able to ensure that the train data and the test data are from completely different subjects. While OpenBMI dataset has EEG signal from 21 subjects, seven-fold cross validation (leave three subjects out) was applied. Thus, the subjects in the testing set were new subjects because they are completely different from the subjects in the training set.

### 3.3. Experiment Settings

In the experiments, the window size *M* was set to 8. In order to divide the input feature vector of length *L* into integer non-overlapping windows, the experiments used a segment of EEG signal of 4.48 s, from 0.5 s before the onset of the MI cue to 3.98 s after the onset of the MI cue, with a total of 1120 sampling points. We first preprocessed the data, bandpass filtered the EEG signals in the range of 4–38 Hz or 0–38 Hz, followed by channel-wise logarithmic sliding normalization, and finally, input the preprocessed EEG signals into the MCL-SWT model. During model training, Adam optimizer [37] was used as the optimization method and the weight decay parameter was set to 0.05. Due to the new experimental setup with new subjects, two categories of three-channel MI classification tasks (C3, Cz, and C4) from dataset IIb were used in this paper. The temporal convolution kernel *T* is set to 25, the spatial convolution kernel *C* is set to 3. The weight coefficients $w_{o}$ and $w_{m}$ are empirically in Equation (12) are set to 0.2 and 0.3, and the weight coefficients α and β are set to 0.2 and 1.2, respectively. The definition of epoch is the number of times the entire dataset is completely traversed (forward and backward) when the model is being trained, and 1 epoch is equal to the number of times all the training samples are learnt by the model. For the IIa and IIb datasets, we train 500 epochs, and for the OpenBMI dataset, we train 400 epochs.

## 4. Results

### 4.1. Performance Comparison of Subject-Independent MI Recogination

This Section evaluates subject-independent MI recognition performance of the MCL-SWT model. Since the subjects in dataset IIa are completely different from the subjects in dataset IIb, we use this cross-dataset setup the evaluate performance of the subject-independent MI based EEG signal recognition. During the training process, the model typically converges within 500 epochs, so the maximum number of training epochs is set to 500 to ensure model’s convergence. While OpenBMI dataset has EEG signal from 21 subjects, seven-fold cross validation (leave three subjects out) was applied. Thus, the subjects in the testing set were new subjects because they are completely different from the subjects in the training set. The maximum number of epochs for the training was set to 500 for dataset 2a and 2b, and 400 for dataset OpenBMI to guarantee convergence of the model. Given the considerable randomness that impacts test accuracy at any given epoch, the evaluation metrics employed in the experiment are as follows: (a) Maximum test accuracy over 500 epochs (Max Accuracy); (b) Average test accuracy from epochs 401 to 500 (Average Accuracy); (c) Test accuracy at the epoch with the lowest training loss (Accuracy). Table 1 presents the results in terms of “Accuracy/Kappa value”, with the best results highlighted in bold. The first row indicates the encoding format as “dataset-bandpass filter”. For example, “2a-0 Hz” indicates that the model was trained on dataset IIb and tested on dataset IIa, preprocessed using a “0–38 Hz” bandpass filter. Five State-of-the-Art models were compared in the experiments, including Shallow ConvNet, Deep ConvNet [16], EEGNet [38], FBCNet [39], and ATCNet [28]. The SWT and MCL-SWT refer to the SWT model without MCL and with MCL, respectively. In addition, Table 2 presents the results of performance comparison on OpenBMI dataset.

A paired-sample one-sided Student’s *t*-test is conducted to confirm the significance of the precision improvement of Table 1 (the null hypothesis is that the accuracy of the MCL-SWT model is equal to the SOTA models, against the alternative that the accuracy of the MCL-SWT model is greater than the accuracy of original model based on different SOTA models). The *p*-values are listed in Table 3.

The experimental results in Table 1, Table 2 and Table 3 lead to the following conclusions: (a) The MCL-SWT model shows an effect in terms of accuracy when compared to several of the compared models. (b) The proposed MCL-SWT model exhibits excellent performance across different datasets and bandpass filters. (c) MCL further improves the recognition accuracy and kappa value of SWT. (d) The proposed SWT model is resistant to overfitting, as evidenced by the small gap between the maximum accuracy/kappa value and the average accuracy/kappa value of SWT. (e) Experimental results show that MCL-SWT has better inter-subject generalization ability. (f) The precision improvement provided by the MCL-SWT is significant.

**Figure 5:**
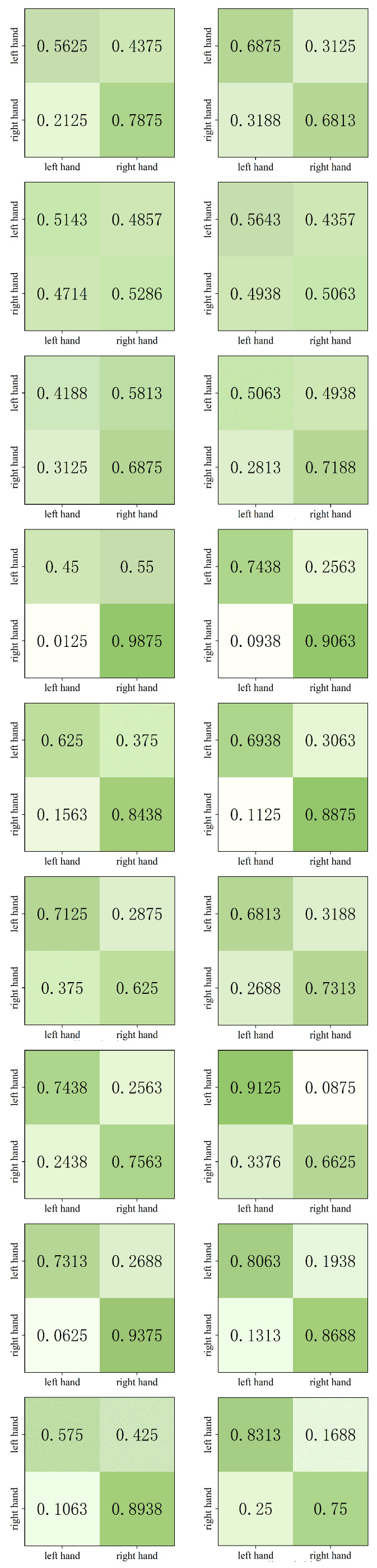
Confusion matrices for SWT (**left**) and MCL-SWT (**right**) across nine subjects in dataset IIb.

To illustrate the classification accuracy across different MI task categories in the MCL-SWT model, Figure 5 presents the confusion matrices for SWT and MCL-SWT across nine subjects in dataset IIb. The first and third rows display the confusion matrices for subjects 1 to 9 (sub1–sub9) using the SWT model, while the second and fourth rows show the confusion matrices for the same subjects using the MCL-SWT model. In each diagram, the horizontal axis represents the predicted classes, and the vertical axis represents the true classes. From these results, we can conclude that: (a) MCL-SWT improves classification accuracy for most subjects compared to SWT. (b) The classification accuracy of MCL-SWT is more balanced across each class.

### 4.2. Sensitivity Analysis of Hyperparameters

The number of temporal multi-head self-attention blocks and the number of heads in each block are two hyperparameters of the SWT model. This subsection investigates the influence of these hyperparameters on the model’s performance, with the experimental results summarized in Table 4. Increasing the number of heads or blocks results in a greater number of model parameters and attention scores, thereby increasing the model’s complexity.

We can draw the following conclusions from Table 4: (a) the performance of the model deteriorates as the number of temporal multi-head self-attention blocks increases—this may be due to the limited number of training samples in MI EEG, which is insufficient to fully train a model with multiple self-attention blocks; (b) The number of self-attention heads has a minimal impact on the model’s performance.

**Figure 6:**
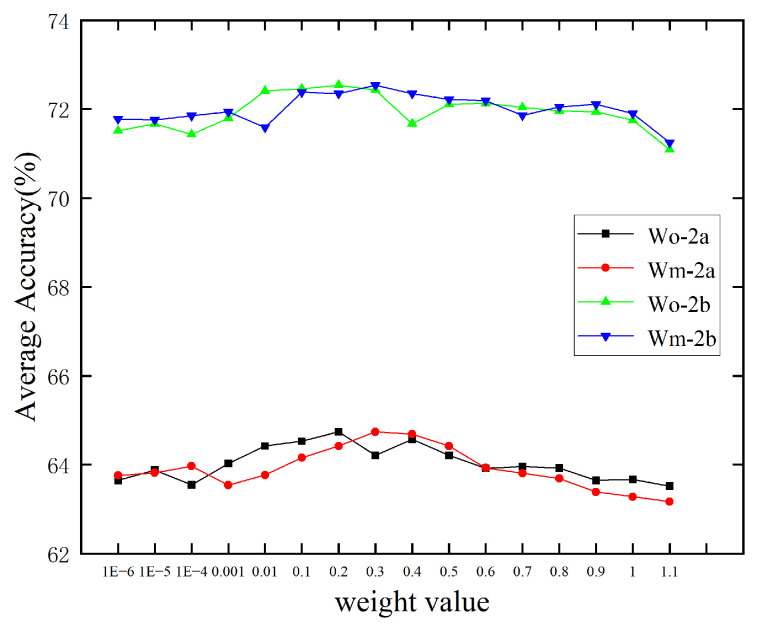
The influence of the hyperparameters $w_{o}$ and $w_{m}$ in the MCL.

We further investigate the impact of the hyperparameters $w_{o}$ and $w_{m}$ in the MCL. Figure 6 illustrates the model’s performance with varying values of $w_{o}$ and $w_{m}$ on datasets IIa and IIb. The preprocessing step involved applying a uniform bandpass filter of “4–38 Hz”. In Figure 6, $W_{m}$-2a represents the experimental results for different values of $w_{m}$ on dataset IIa with $w_{o}$ fixed, while $W_{o}$-2b presents the experimental results for varying $w_{o}$ on dataset IIb with $w_{m}$ fixed. The results suggest that values within the range [0.1,0.3] are effective for both datasets.

α and β are other two hyperparameters in the MCL training process. In the experiments, α and β were assigned different values, and their impact on the method’s performance was evaluated. The specific experimental results are summarized in Table 5. Using a “4–38 Hz” bandpass filter for preprocessing, we examined the effect of various hyperparameter values on the method’s performance by training the model on dataset IIa and testing it on new subjects from dataset IIb. The experimental results in Table 5 demonstrate the model’s robustness to variations in hyperparameters.

### 4.3. Feature Visualization

**Figure 7:**
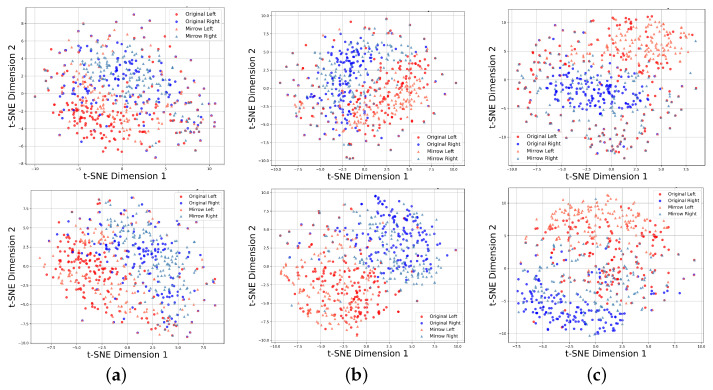
Feature distribution visualization of SWT without MCL (**top**) and with MCL (**bottom**) on datasets IIb. (**a**) Feature distribution of Subject 4. (**b**) Feature distribution of Subject 5. (**c**) Feature distribution of Subject 8.

To assess the impact of MCL on feature distribution, the features were visualized using t-SNE [40], with the results for subjects 4, 5, and 8 from dataset IIb shown in Figure 7. In these visualizations, the original EEG signals are represented as circles, while the mirror EEG signals are depicted as triangles. The left-hand MI is shown in red, and the right-hand MI is shown in blue.

From Figure 7, it is evident that MCL effectively separates features from different MI tasks and clusters features from the same MI task. Without MCL, the feature distributions of the left-hand and right-hand MI tasks exhibit significant overlap, making it challenging to distinguish between them. However, with MCL applied, the features from the same task (e.g., left-hand MI) are grouped more tightly, while features from opposite tasks are pushed further apart. This demonstrates the ability of MCL to enhance feature separability by leveraging mirror EEG data to align positive samples and enforce the distinction between negative sample pairs. By incorporating mirror EEG signals, MCL also enhances the model’s ability to localize ERD/ERS phenomena accurately. This approach facilitates improved recognition of subject-independent MI EEG signals, confirming the effectiveness of MCL-SWT in addressing challenges associated with individual variability in EEG feature distributions.

### 4.4. Ablation Experiment on Mirror Contrastive Loss

**Figure 8:**
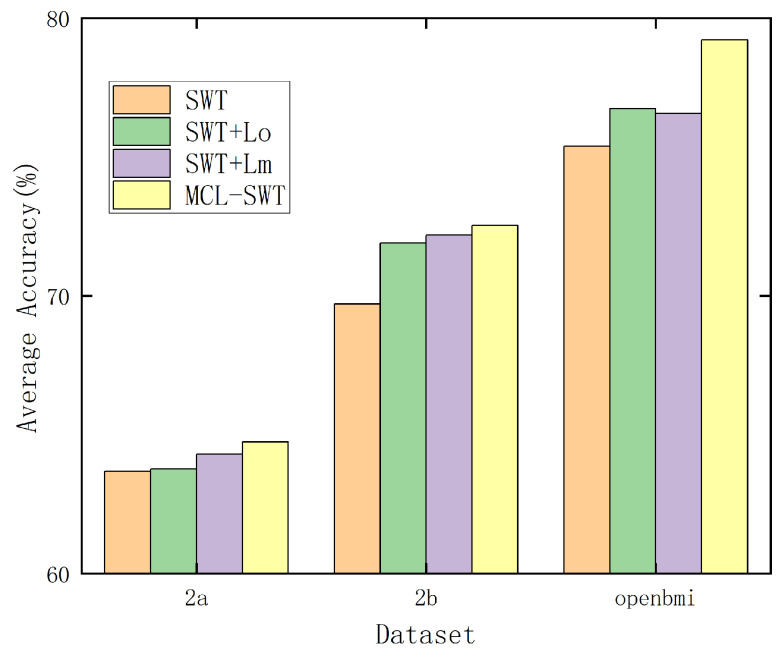
Ablation experiments of $L_{o}$ and $L_{m}$.

Equation (12) consists of two components: the first term, $L_{o}$, represents the contrastive loss within the original EEG signals, while the second term, $L_{m}$, corresponds to the contrastive loss between the original EEG signals and their mirror counterparts. To evaluate the effectiveness of each component, ablation experiments were conducted on datasets IIa and IIb, with the results presented in Figure 8. For preprocessing, a “4–38 Hz” band-pass filter was applied. As shown in Figure 8, both $L_{o}$ and $L_{m}$ individually contribute to improving the average classification accuracy of the SWT model. Moreover, the combination of $L_{o}$ and $L_{m}$ further enhances the model’s performance, demonstrating the complementary benefits of incorporating both components.

To assess the performance across different subjects, Table 6, Table 7 and Table 8 present the results of our proposed MCL method for each subject on datasets IIa, IIb and OpenBMI. Uniform preprocessing using a “4–38 Hz” bandpass filter on datasets IIa and IIb. Preprocessing method using the “8–30 Hz” bandpass filter on the OpenBMI dataset. Table 8 present the results of our proposed MCL method for each subject on OpenBMI datasets. A uniform preprocessing approach using a “8–30 Hz” bandpass filter was applied. The results indicate that the impact of each component varies across individual subjects. However, on average, both components contribute to performance improvement. Furthermore, the combination of $L_{o}$ and $L_{m}$ provides additional performance gains, consistent with the findings illustrated in Figure 8.

### 4.5. Computing Complexity Analysis

The number of model parameters and inference time are key indicators of computational complexity and were evaluated in this section. Inference time was measured as the average duration of 1000 runs. All experiments were conducted on a server equipped with an Intel i7 10700K CPU and an NVIDIA GeForce RTX 3090 GPU. The results of the analysis are presented in Table 9, which shows that the MCL-SWT model achieves a good balance between parameter size and inference efficiency. While it has more parameters (155 M) than lightweight models like EEGNet and FBCNet (3 M), its inference time (8.36 ms) is significantly faster than FBCNet (37.64 ms) and ATCNet (15.37 ms). This highlights MCL-SWT’s efficiency in maintaining high performance with low computational overhead, which shows the potential to be applied in online BCI system.

## 5. Discussion

The utility of a brain-computer interface system is inextricably linked to the performance of its decoding module. For subject-independent EEG signal recognition, the variability of ERD/ERS spatial patterns across individuals poses a significant challenge to EEG signal decoding. We propose a mirror-contrastive learning-based sliding window transformer (MCL-SWT) to improve the spatial sensitivity of ERD/ERS by introducing mirror EEG signals and contrastive learning, and also to deal with inter-subject variability effectively. Additionally, we introduce the SWT mechanism to reduce the computational complexity compared to the global attention approach. Therefore, the proposed method improves subject-independent MI-based EEG signal recognition compared to existing models [16,28,38,39].

In the experiments, we can see that MCL-SWT achieves good results on the three datasets. The temporal SWT mechanism computes self-attention scores within a sliding window, effectively balancing improved model performance with acceptable computational complexity. We explored the impact of several key parameters on the model and showed that increasing the number of temporal multi-head self-attention blocks and the number of heads in each block leads to more model parameters and attention scores, increasing the complexity of the model and thus affecting classification performance. We also performed ablation experiments and feature visualizations where features from the same task (e.g., left MI) were more tightly grouped and features from the opposite task were pushed further apart with the application of MCL. The ability of MCL to enhance feature separability by using mirrored EEG data to align positive samples and force the distinction between negative sample pairs is demonstrated, enhancing the model’s ability to accurately localise ERD/ERS phenomena. In the BCI field, where existing technologies are already quite mature, the room for enhancing the performance of high-precision algorithms and systems is inherently limited. Consequently, while the 2.82% and 2.17% accuracy improvements may appear modest numerically, from a practical application standpoint, these enhancements enable the system to significantly reduce misidentifications across extensive sample datasets.

However, although the SWT mechanism reduces computational complexity compared to global attention methods, its scalability to large-scale datasets or real-time applications requires further study. Future research could also focus on optimizing MCL-SWT for multi-class MI tasks or exploring its application in hybrid brain-computer interface (BCI) systems. Furthermore, most motor imagery EEG recognition methods are evaluated on EEG signals from healthy subjects, but motor imagery brain-computer interfaces are valuable in neurorehabilitation (e.g., post-stroke rehabilitation) [6,7]. Therefore, future research is planned on MI-EEG recognition algorithms for stroke patients.

Lastly, combining MCL with domain adaptation or transfer learning could further enhance its ability to generalize across subjects and even across datasets, paving the way for broader applicability in BCI applications.

## 6. Conclusions

In this paper, we propose a Mirror Contrastive Learning (MCL) method combined with a Sliding Window Transformer (SWT) model for subject-independent EEG signal recognition. By constructing mirror EEG signals, the model’s sensitivity to the spatial location of ERD/ERS phenomena is enhanced. In addition, the temporal SWT mechanism computes self-attention scores within a sliding window, effectively balancing improved model performance with acceptable computational complexity. Detailed comparative experiments on different EEG datasets have been conducted with promising results. Furthermore, MCL, as a generalizable approach, has the potential to be integrated into various backbone networks for MI-EEG recognition.

## Figures and Tables

**Table 1:** Classification performance (Accuracy and Kappa value) of subject-independent MI recognition on dataset 2a and 2b. The best results are highlighted in bold.

		2a-0 Hz	2a-4 Hz	2b-0 Hz	2b-4 Hz
Avg Acc/ Kappa	Shallow	63.72/0.29	60.76/0.25	72.70/0.46	67.06/0.34
	Deep	60.55/0.25	60.80/0.25	71.55/0.44	65.93/0.32
	EEGNet	60.58/0.25	59.52/0.23	71.21/0.44	65.91/0.32
	FBCNet	60.56/0.25	59.73/0.23	70.55/0.43	65.29/0.31
	ATCNet	58.87/0.22	57.45/0.20	68.47/0.36	64.69/0.30
	SWT	66.00/0.33	63.68/0.28	74.50/0.49	69.71/0.40
	MCL-SWT	**66.56/0.33**	**64.74/0.30**	**75.85/0.52**	**72.54/0.45**
Acc/ Kappa	Shallow	63.66/0.28	61.18/0.26	73.45/0.47	68.70/0.36
	Deep	61.27/0.26	61.34/0.26	72.89/0.46	66.51/0.34
	EEGNet	61.19/0.26	58.72/0.22	72.24/0.45	66.20/0.33
	FBCNet	60.86/0.25	58.36/0.22	71.18/0.44	65.53/0.31
	ATCNet	59.34/0.23	57.78/0.20	69.13/0.39	63.78/0.27
	SWT	66.13/0.33	63.58/0.28	74.58/0.49	69.79/0.40
	MCL-SWT	**66.48/0.33**	**64.52/0.30**	**75.62/0.51**	**73.27/0.47**
Max Acc/ Kappa	Shallow	67.28/0.34	65.36/0.32	75.18/0.51	71.69/0.45
	Deep	66.05/0.33	65.35/0.32	73.73/0.48	71.68/0.45
	EEGNet	65.51/0.32	64.51/0.30	73.31/0.47	71.16/0.44
	FBCNet	66.46/0.33	63.89/0.29	72.93/0.46	70.98/0.43
	ATCNet	64.11/0.29	62.18/0.28	70.84/0.43	68.22/0.36
	SWT	66.82/0.34	64.81/0.30	75.49/0.51	74.12/0.48
	MCL-SWT	**67.37/0.35**	**65.49/0.31**	**76.37/0.53**	**75.49/0.51**

**Table 2:** Classification performance (Accuracy and Kappa value) of subject-independent MI recognition on OpenBMI dataset. The best results are highlighted in bold.

		Shallow	Deep	EEGNet	FBCNet	ATCNet	MCL-SWT
Avg Acc/ Kappa	Fold1	81.61/0.63	79.56/0.59	80.17/0.60	79.00/0.58	80.89/0.62	82.56/0.65
	Fold2	77.67/0.55	75.89/0.52	75.94/0.52	71.56/0.43	73.72/0.47	80.01/0.60
	Fold3	78.33/0.57	80.44/0.61	82.00/0.64	82.39/0.65	83.83/0.68	76.38/0.53
	Fold4	81.00/0.62	81.22/0.62	82.06/0.64	79.11/0.58	80.06/0.60	85.51/0.71
	Fold5	74.39/0.49	72.28/0.45	76.89/0.54	71.67/0.43	72.94/0.46	75.48/0.51
	Fold6	80.33/0.61	82.61/0.65	82.75/0.66	76.89/0.54	81.78/0.64	80.46/0.61
	Fold7	70.33/0.41	69.22/0.38	70.44/0.41	67.83/0.36	72.28/0.45	74.05/0.48
	Avg	77.67/0.55	77.32/0.55	78.61/0.57	75.49/0.51	77.93/0.56	**79.21/0.58**

**Table 3:** The paired-sample one-sided Student’s *t*-test of the performance on Table 1.

MCL-SWT vs.	Shallow	Deep	EEGNet	FBCNet	ATCNet
*p*-Values	0.0036	0.0020	0.0005	0.0007	0.0001

**Table 4:** The influence of the head number of self-attention and the number of multi-head self-attention block on the accuracy. The best results are highlighted in bold.

	4 Heads	8 Heads	10 Heads
1 block	**74.71/0.49**	**74.50/0.49**	**74.73**/**0.49**
2 block	74.30/0.48	74.20/0.48	74.45/0.49
3 block	73.16/0.46	73.67/0.47	73.26/0.46

**Table 5:** The performance evaluation on different hyperparameters in MCL. The best results are highlighted in bold.

hyperparameter values	β=1.2		
	α=0.2	α=0.4	α=0.8
Average Accuracy	**72.54**	72.29	72.26
hyperparameter values	α=0.2		
	β=0.6	β=1.2	β=2.4
Average Accuracy	71.95	**72.54**	72.02

**Table 6:** Ablation experiments of $L_{o}$ and $L_{m}$ for each subject on dataset IIa. The best results are highlighted in bold. The check mark means the related loss is included.

SWT	$L_{o}$	$L_{m}$	sub1	sub2	sub3	sub4	sub5	sub6	sub7	sub8	sub9	Avg
✓			66.53	54.39	88.65	58.92	53.87	57.51	**62.35**	71.52	**59.39**	63.68
✓	✓		66.72	**58.73**	88.77	58.01	54.31	56.82	60.34	**74.03**	56.19	63.77
✓		✓	67.76	58.33	**89.97**	57.89	55.37	58.51	61.58	72.33	56.97	64.30
✓	✓	✓	**68.81**	57.26	89.83	**59.67**	**56.95**	**59.85**	59.18	73.18	57.94	**64.74**

**Table 7:** Ablation experiments of $L_{o}$ and $L_{m}$ for each subject on dataset IIb. The best results are highlighted in bold. The check mark means the related loss is included.

SWT	$L_{o}$	$L_{m}$	sub1	sub2	sub3	sub4	sub5	sub6	sub7	sub8	sub9	Avg
✓			65.83	53.12	54.75	80.23	73.74	67.60	73.30	83.50	75.32	69.71
✓	✓		**69.61**	54.15	55.50	81.63	78.71	68.70	**75.70**	86.23	76.85	71.90
✓		✓	68.36	53.97	56.76	**82.82**	**78.84**	**70.53**	73.76	85.54	**79.09**	72.19
✓	✓	✓	69.50	**55.69**	**57.13**	82.71	78.17	69.86	74.75	**86.93**	78.13	**72.54**

**Table 8:** Ablation experiments of $L_{o}$ and $L_{m}$ for each subject on OpenBMI dataset. The best results are highlighted in bold. The check mark means the related loss is included.

SWT	$L_{o}$	$L_{m}$	Fold1	Fold2	Fold3	Fold4	Fold5	Fold6	Fold7	Avg
✓			78.00	75.61	74.71	76.43	73.31	78.11	71.56	75.39
✓	✓		80.93	76.90	75.66	78.05	74.02	79.31	72.38	76.75
✓		✓	80.68	77.74	75.61	77.98	74.25	78.28	71.35	76.56
✓	✓	✓	**82.56**	**80.01**	**76.38**	**85.51**	**75.48**	**80.46**	**74.05**	**79.21**

**Table 9:** Model complexity analysis.

	Shallow	Deep	EEGNet	FBCNet	ATCNet	MCL-SWT
Para num (M)	10	268	3	3	37	155
Infer time (ms)	0.56	1.42	2.48	37.64	15.37	8.36

## Data Availability

In this study, we utilized publicly available datasets. These datasets can be accessed at: 25 April 2025 https://www.bbci.de/competition/download/competition_iv/BCICIV_2a_gdf.zip, https://www.bbci.de/competition/download/competition_iv/BCICIV_2b_gdf.zip and https://gigadb.org/dataset/view/id/100542/File_page/5.
